# Primary plasmacytoma of the larynx: A case report and literature review

**DOI:** 10.1097/MD.0000000000045040

**Published:** 2025-10-17

**Authors:** Yu-Xin Xia, Zhong-Shan Luo, Ming-Hui Wang, Lian-He Yang, Endi Wang

**Affiliations:** a Department of Pathology, First Affiliated Hospital and College of Basic Medical Sciences, China Medical University, Shenyang, Liaoning, China; b Department of Surgery, Datong County People’s Hospital of Qinghai Province, Qinghai, China; c Department of Pathology, Keck School of Medicine, University of Southern California, Los Angeles, CA.

**Keywords:** diffuse large B-cell lymphoma, larynx, lymphoma, plasmacytoma, prognosis

## Abstract

**Rationale:**

Primary laryngeal plasmacytoma is a rare form of extramedullary plasma cell neoplasm. By definition, it is confined to the larynx without involvement of the bone marrow or peripheral blood. To our best knowledge, there are only 51 cases reported in English literature. We report a case of primary plasmacytoma of the larynx, focusing on the clinical features, pathological diagnosis, treatment, and prognosis of this rare entity.

**Patient concerns:**

The patient is a 64-year-old Asian male who presented with hoarseness and a sensation of a foreign body in the throat.

**Diagnoses:**

Laryngoscopy revealed a gray-white mass in the left laryngeal ventricle, raising suspicion of a pathological lesion and prompting a biopsy for diagnostic evaluation. Histopathological examination demonstrated a diffuse, invasive proliferation of abnormal plasma cells. Immunohistochemical analysis showed diffuse positivity for CD138 and exhibited restricted expression of kappa light chain in plasma cells. Based on the morphological and immunohistochemical findings, a diagnosis of primary plasmacytoma of the larynx was established.

**Interventions:**

The patient underwent radiotherapy treatment.

**Outcomes:**

During 1 year of follow-up, no signs of recurrence or progression were observed.

**Lessons:**

Given its rarity, primary plasmacytoma of the larynx must be distinguished from plasma cell myeloma involving the larynx, certain types of lymphoma, malignant melanoma, and myoepithelial carcinoma. Pathological examination remains essential for accurate diagnosis.

## 1. Introduction

Plasmacytoma is a malignant tumor of the hematological system arising from clonal proliferation of plasma cells. According to the 5th edition of World Health Organization classification, plasmacytomas are categorized into solitary plasmacytoma of bone and extramedullary plasmacytoma.^[1]^ Extramedullary plasmacytoma refers to a localized plasma cell neoplasm occurring in tissue outside the bone or bone marrow and accounts for approximately 3% to 5% of all plasmacytomas. Notably, about 80% of extramedullary plasmacytomas arise in lymphoid-rich tissues of the upper respiratory tract, including the oropharynx, nasopharynx, and paranasal sinuses.^[2]^ However, primary plasmacytoma of the larynx is exceedingly rare, with only about 51 cases reported to date (Table 1).^[3–45]^ Among these, the epiglottis is identified as the most common site of involvement, followed by the vocal cords and false vocal cords.^[55]^ The diagnosis of primary laryngeal plasmacytoma requires confirmation of disease localized to the larynx, with no evidence of bone marrow and peripheral blood involvement.^[56]^ Therefore, comprehensive radiological imaging and laboratory evaluations are essential to exclude systemic disease and confirm the diagnosis as a distinct type of extramedullary plasmacytoma.^[57]^ Interestingly, the clinical behavior and prognosis of primary plasmacytoma of the larynx appear to differ from those of plasmacytoma arising in other tissue, as suggested by recent literatures.^[58,59]^ Herein, we report a case of primary plasmacytoma of the larynx in a 64-year-old Asian male and provide a review of the literature, focusing on the clinical features, pathological diagnosis, treatment, and prognosis of this rare entity. The clinical characteristics of differential diagnosis for this disease were also listed, providing a basis for the correct diagnosis of this disease (Table 2).^[46–54]^

**Table 1:** Literature review of case reports of primary plasmacytoma of the larynx.

Case	Publication year	Age	Gender	Clinical manifestation	Treatment	Outcomes
Barbu et al^[3]^	1992	69	Male	Hoarseness and dysphagia	Radiotherapy	Disease-free survival
Mochimatsu et al^[4]^	1993	54	Male	Globus sensation	Radiotherapy	Disease-free survival
Weissman et al^[5]^	1993	76	Male	Hoarseness	Surgical resection and radiotherapy	Disease-free survival
Zbären et al^[6]^	1995	88	Male	Hoarseness	Untreated	Disease-free survival
Zbären et al^[6]^	1995	71	Male	Hoarseness	Untreated	Disease-free survival
Nowak et al^[7]^	1998	34	Male	Hoarseness	Radiotherapy	NA
Nowak et al^[7]^	1998	50	Male	Hoarseness	Radiotherapy	NA
Nowak et al^[7]^	1998	36	Male	Asymptomatic	Radiotherapy	NA
Nowak et al^[7]^	1998	68	Female	Hoarseness	Radiotherapy	NA
Nowak et al^[7]^	1998	48	Male	Hoarseness	Radiotherapy	NA
Welsh et al^[8]^	1998	59	Male	Dry cough and dyspnea	Radiotherapy	Disease-free survival
Rakover et al^[9]^	2000	38	Male	Hoarseness	Surgical resection and radiotherapy	Disease-free survival
Nagasaka et al^[10]^	2001	12	Female	Hoarseness	Radiotherapy	Disease-free survival
Kamijo et al^[11]^	2002	84	Male	Hoarseness	Radiotherapy	Disease-free survival
Soni et al^[12]^	2002	65	Male	Hoarseness	Radiotherapy	Disease-free survival
Nakashima et al^[13]^	2006	39	Male	Asymptomatic	Surgical resection and radiotherapy	Disease-free survival
Nakashima et al^[13]^	2006	59	Male	Asymptomatic	Surgical resection	Disease-free survival
Velez et al^[14]^	2007	64	Male	Hoarseness	Surgical resection and radiotherapy	Disease-free survival
Kusunoki et al^[15]^	2007	76	Female	Asymptomatic	Untreated	NA
Ozbilen Acar et al^[16]^	2008	43	Female	Hoarseness	Surgical resection	Disease-free survival
Grabowska et al^[17]^	2008	46	Female	Hoarseness	Untreated	Progressed to squamous cell carcinoma
Cb et al^[18]^	2009	49	Male	Hoarseness	Radiotherapy	Disease-free survival
Vanan et al^[19]^	2009	16	Female	Hoarseness and dysphagia	Radiotherapy	Disease-free survival
Pratibha et al^[18]^	2009	49	Male	Hoarseness	Radiotherapy	Disease-free survival
Rutherford et al^[20]^	2009	13	Female	Dysphonia	Surgical resection and radiotherapy	NA
Zhang et al^[21]^	2010	56	Female	Hoarseness	Surgical resection	Disease-free survival
Pichi et al^[22]^	2011	73	Male	Hoarseness and dyspnea	Radiotherapy	Progressed to multiple myeloma
Kim et al^[23]^	2011	58	Male	Asymptomatic	Surgical resection	Disease-free survival
González Guijarro et al^[24]^	2011	11	Male	Dysphonia	Surgical resection and radiotherapy	Disease-free survival
Pinto et al^[25]^	2012	49	Female	Dysphonia	Surgical resection	Disease-free survival
Ramírez-Anguiano et al^[26]^	2012	57	Male	Hoarseness and dysphagia	Radiotherapy	Disease-free survival
Ravo et al^[27]^	2012	56	Male	Hoarseness and dysphagia	Radiotherapy	Disease-free survival
De Zoysa et al^[28]^	2012	62	Female	Hoarseness	Radiotherapy	Disease-free survival
Ghatak et al^[29]^	2013	29	Female	Hoarseness	Radiotherapy	Disease-free survival
Loyo et al^[30]^	2013	80	Female	Hoarseness	Surgical resection	NA
Wang et al^[31]^	2015	43	Male	Hoarseness and dyspnea	Surgical resection	Progressed to multiple myeloma
Xing et al^[32]^	2015	47	Female	Hoarseness	Radiotherapy	Disease-free survival
Pino et al^[33]^	2015	65	Male	Dysphonia	Radiotherapy	NA
Wang et al^[31]^	2015	43	Male	Hoarseness	Untreated	Progressed to multiple myeloma
Krebs et al^[34]^	2017	77	Male	Dyspnea and globus sensation	Radiotherapy	Disease-free survival
Ge et al^[35]^	2018	46	Male	Cough and sore throat	Radiotherapy and chemotherapy	Progressed to AML
Du et al^[36]^	2019	42	Male	Hoarseness and cough	Surgical resection	Disease-free survival
Brandt et al^[37]^	2020	54	Female	Dysphonia	Radiotherapy	Disease-free survival
Čunović et al^[38]^	2020	68	Male	Hoarseness and dyspnea	Radiotherapy	Disease-free survival
Ergun et al^[39]^	2022	66	Male	Dysphagia and dysgeusia	Radiotherapy	Disease-free survival
Lu et al^[40]^	2022	57	Female	Hoarseness	Surgical resection and radiotherapy	Disease-free survival
Shankar et al^[41]^	2022	46	Male	Hoarseness	Radiotherapy	Disease-free survival
Tanrivermis Sayit et al^[42]^	2022	74	Female	Hoarseness and dysphagia	Radiotherapy and chemotherapy	NA
Miyamori et al^[43]^	2024	72	Male	Dysphagia	Surgical resection	Disease-free survival
Hodroj et al^[44]^	2024	38	Female	Hoarseness and dysphonia	Surgical resection	Disease-free survival
Gupte et al^[45]^	2024	77	Male	Hoarseness	Radiotherapy	Disease-free survival
This report	2025	64	Male	Hoarseness	Radiotherapy	Disease-free survival

AML = acute myeloid leukemia, NA = not available.

**Table 2:** Clinically, symptoms of primary laryngeal plasmacytoma are nonspecific and must be differentiated from several other entities.

Differential diagnosis	Median age (yr)	Male/female ratio	Primary site	Clinical characteristics	Histopathology	Immunohistochemistry	Genetic studies	Treatment
Plasma cell myeloma involving larynx^[46,47]^	64	4.6:1	Epiglottis	Plasma cell myeloma typically involves widespread bone marrow infiltration, often producing monoclonal immunoglobulin detectable in serum and/or urine.	Infiltration of plasma cells with consistent size.	B lymphocytes and plasma cells for the CD138 marker.	Point mutations of the N-ras and K-ras genes, as well as mutations or deletions of the tumor suppressor genes p53 and Rb-1.	Surgery for local excision of tumor masses followed by radiotherapy was recommended.
Plasmablastic lymphoma^[48,49]^	HIV positive is 46 and HIV negative is 57	HIV-positive patients is 8/1 HIV-negative patients is 1.7–1.9/1	The oral cavity and/or gastrointestinal tract	A highly aggressive variant of diffuse large B-cell lymphoma, typically seen in immunocompromized patients (e.g., HIV, transplant recipients).	Large plasmacytoid cells resembling immunoblasts, characterized by prominent nucleoli, and abundant cytoplasm.	PBL and plasmacytoma share immunophenotypic features.	EBV latent infection and MYC gene rearrangement.	CHOP
Plasma cell granuloma^[50]^	52	1:2	Lungs	Plasma cell granuloma is a benign, polyclonal proliferation of plasma cells, typically associated with fibrosis.	Sheets, aggregates, and infiltrating groups of plasma cells with intervening fibrous stroma and minimal to absent myofibroblastic proliferation.	No malignant B cell population and a normal κ-λ ratio.	A disorder of excessive polyclonal proliferation of plasma cells.	Surgery
MALT lymphoma with extensive plasmacytic differentiation^[51]^	65	1:1.5	Gastrointestinal tract	MALT lymphoma is classified as a low-grade B-cell lymphoma with a relatively good prognosis. Most patients are asymptomatic at the time lymphoma been diagnosed.	Lymphocyte focal aggregation with extensive plasma cell differentiation, and reactive lymphoid follicles with germinal centers.	Positive for CD20, CD79a, BCL-2, CD138, lambda light chain, and focally positive for CD43, but negative for CD3, CD5, CD23, CD10, CD21, CyclinD1, BCL-6, and kappa light chain.	Immunoglobulin gene rearrangements and the API2-MALT1 fusion gene.	Surgical resection is reserved for localized tumors, and chemotherapy is given for bilateral or diffuse involvement.
Malignant melanoma^[52]^	70	1.5:1	Supraglottic larynx	Primary mucosal melanoma of the upper airways and digestive tract is associated with a very poor prognosis.	Marked atypia with multinucleated epithelioid polygonal cells admixed with spindle cells was seen.	Positivity for Melan-A, S-100 protein, HMB-45 and vimentin.	BRAF mutation	Surgery
Myoepithelial carcinoma^[53,54]^	50	4:1	Supraglottic larynx	Clinical manifestations include hoarseness and dysphagia to solids.	Cells with plasmocytoid appearance against a myxoid background.	Cytokeratin, vimentin, and myoepithelial stains (such as smooth muscle actin, P63, or S-100 protein) are positive.	The abnormality of chromosome 8.	Wide surgery resection

EBV = Epstein–Barr virus, HIV = human immunodeficiency virus, MALT = mucosa-associated lymphoid tissue, MYC = myelocytomatosis oncogene, PBL = plasmablastic lymphoma.

## 2. Case presentation

**Figure 1.**
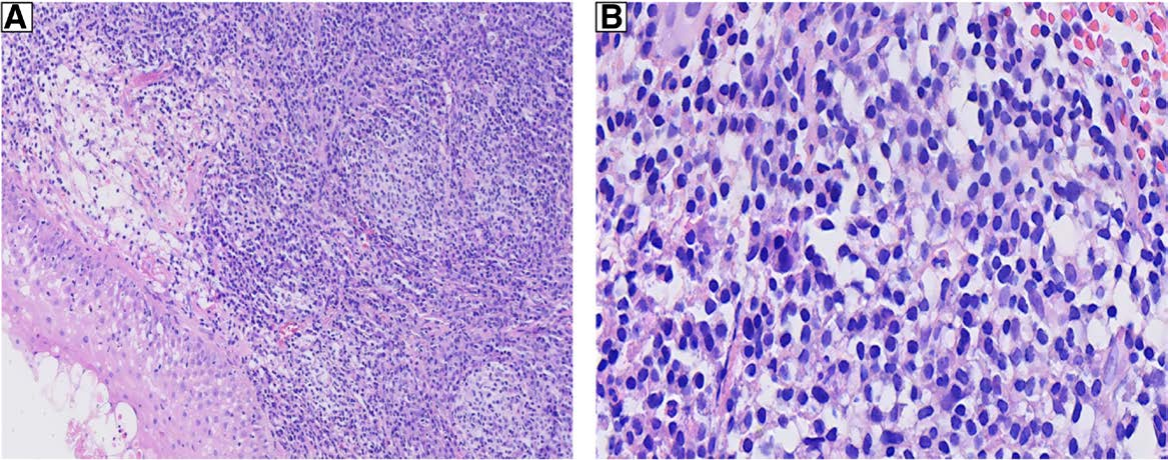
(A) Under squamous epithelium, the neoplastic cells show diffuse proliferation forming a confluent growth pattern. Mild vascular proliferation was observed, and mitotic figures were rare (H&E, 100×). (B) At high magnification, the tumor cells were relatively single, with plasma cell-like morphology, rough chromatin, abundant cytoplasm, eccentric nucleus, round or oval shape, and obvious nucleoli (H&E, 400×).

**Figure 2.**
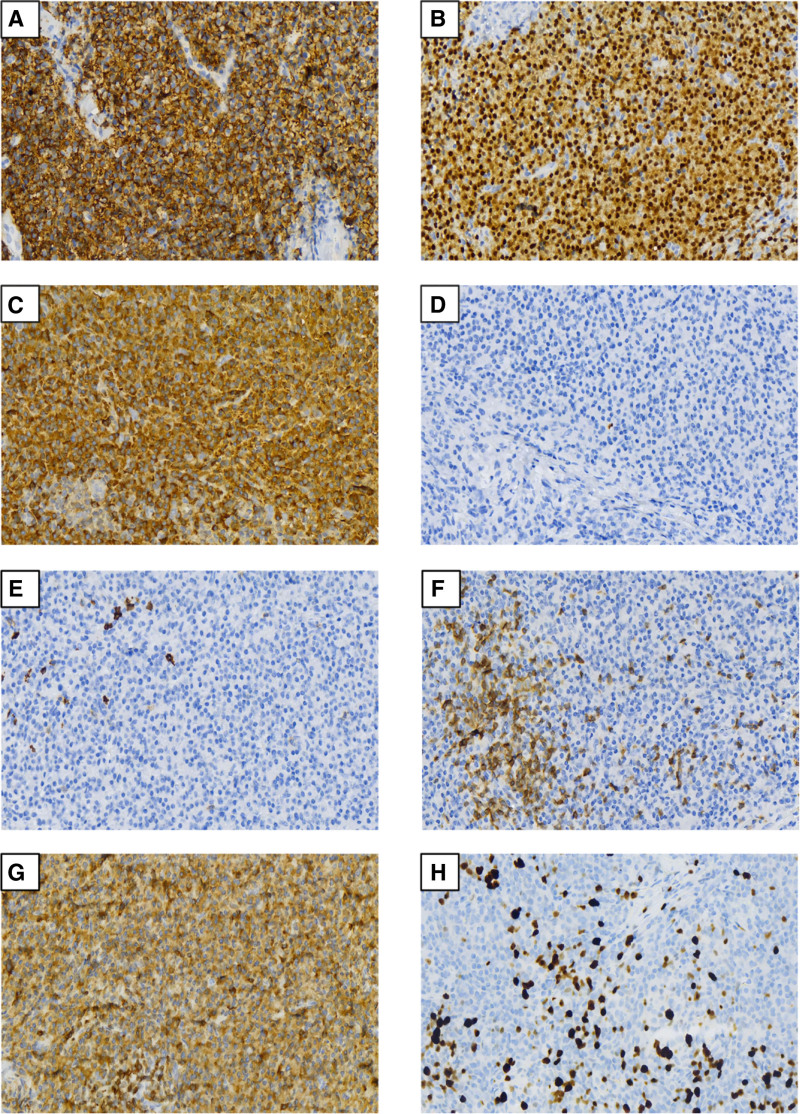
(A) Immunohistochemical staining show membranous positivity for CD138 in the neoplastic cells (200×); (B) positive for MUM1 in the neoplastic cells (200×); (C) positive for kappa light chain (200×); (D) negative for lambda light chain (200×); (E) CD20 stain is negative in the plasmacytoid cells (200×); (F) CD3 stain highlights scattered reactive T-cells; neoplastic cells are negative for the stain (200×); (G) BCL2 stain is positive in the plasmacytoid cells (200×); (H) Ki-67 stain demonstrates a proliferation index of approximately 20% of the total mononucleated cells (200×).

A 64-year-old Asian male presented to our hospital with complaints of hoarseness, a foreign body sensation in the throat, and intermittent nocturnal dyspnea. The patient denied other significant symptoms and had no history of hypertension, diabetes, coronary heart disease, and other relevant medical conditions. There was no family history of malignancy. Laryngoscopic examination revealed a narrow, gray-white nodular protrusion at the base of the left laryngeal ventricle. The nodule, measuring approximately 1 cm in diameter, had a smooth surface, and vocal cord mobility was preserved. No abnormalities were detected in the nasopharynx or oropharynx, aside from changes consistent with chronic inflammation. Superficial regional lymphadenopathy was not identified on palpation. The laryngeal nodule was surgically resected for histopathological evaluation. On H&E staining, the specimen demonstrated squamous epithelium-lined mucosa with diffuse proliferation of plasmacytoid cells within the subepithelial stroma. These plasmacytoid cells appeared slightly larger than normal plasma cells, displaying cytological atypia, including centrally placed nuclei. The morphologic features were suggestive of plasma cell neoplasm (Fig. 1). Immunohistochemical analysis revealed that the plasmacytoid cells were positive for CD138, MUM1, and BCL2 and partially positive for CD56. The neoplastic cells exhibitted kappa light chain restriction and showed a Ki-67 proliferation index of 20% (Fig. 2). Additional markers, including cytokeratin, CD3, BCL6, CD21, CD10, and Epstein–Barr virus-encoded RNA by chromogenic in situ hybridization were negative in the plasmacytoid cells (Fig. 2). These morphological and immunohistochemical findings supported the diagnosis of a plasma cell neoplasm. For a more comprehensive examination, the patient underwent an 18-fluorodeoxyglucose positron emission tomography-computed tomography imaging scan, which showed localized and increased fluorodeoxyglucose uptake within the left laryngeal ventricle (SUVmax = 7.8), with no abnormalities in the bone or other tissues. The staging bone marrow biopsy was negative for involvement by a plasma cell neoplasm. It is worth mentioning that the patient tested negative for human immunodeficiency virus (HIV). Based on clinical experience, we will not consider HIV-associated diseases at this time, such as plasmablastic lymphoma, which is often associated with HIV infection. Thereafter, a diagnosis of primary plasmacytoma of the larynx was established.

## 3. Discussion

Pathological examination is crucial for the diagnosing primary plasmacytoma of the larynx. Grossly, these tumors typically present as a pedunculated or broad-based mass or polypoid nodule, often dark red in color and resembling normal laryngeal mucosa. In some cases, the tumors may appear white. The morphology can vary, exhibiting a smooth or irregular surface. The tumor may compress adjacent structures, potentially leading to airway stenosis or obstruction. Occasionally, laryngeal plasmacytoma can cause ulceration and erosion, resulting in laryngeal bleeding. Histologically, primary laryngeal plasmacytoma shares features with plasmacytoma arising in other anatomic locations. Typically, these tumors are well-differentiated, and at low magnification, neoplastic plasma cells are arranged in focal, nodular, fascicular, or sheet-like pattern within the subepithelial stroma of squamous mucosa. At high magnification, the neoplastic cells exhibit condensed nuclear chromatin with inconspicuous nucleoli, and amyloid deposits are rarely observed.^[56]^ Some reports describe poorly differentiated cases, in which neoplastic cells exhibit pleomorphic nuclei. Susnerwala et al proposed that the degree of cellular atypia and nuclear pleomorphism, as markers of histological grades, correlate with tumor aggressiveness.^[60]^ Low-grade plasmacytomas often respond well to regional radiotherapy, whereas those with intermediate or high-grade plasmacytoma are more challenging to control. Classically, plasmacytomas display plasmacytoid cells with abundant cytoplasm and eccentric nuclei with clumped chromatin. However, in some cases, immature cells with scant cytoplasm and central nuclei with disperse chromatin may be prominent.^[61]^ These poorly differentiated cases, especially in uncommon sites like the larynx, present significant diagnostic challenges. Although histological grading assists in diagnosis, confirmation of light chain restriction (kappa or lambda) is essential to establish the neoplastic nature of the plasma cells. Mock et al demonstrated that in extramedullary plasmacytoma of the head and neck, the most common immunoglobulin expressed is IgG with κ light chain restriction and that these cases have the lowest rate of progression to plasma cell myeloma. In contrast, cases with λ light chain restriction often exhibit immature morphology and are more prone to progression.^[62]^ Molecular studies frequently reveal clonal rearrangement of IGH and/or IGK genes, further supporting the diagnosis. The diagnostic criteria for primary plasmacytoma of the larynx include:

1. Histological confirmation of a plasma cell neoplasm in a solitary laryngeal lesion.
2. Normal bone marrow biopsy, with no clonal or abnormal plasma cells.
3. Absence of systemic disease on imaging (magnetic resonance imaging or computed tomography) or laboratory evaluation.
4. No evidence of end-organ damage attributable to plasma cell proliferative disease.

The mainstay of treatment for localized laryngeal plasmacytoma is surgical resection and/or radiotherapy. Radiotherapy is considered first-line therapy, essentially for tumors < 5 cm in diameter.^[63]^ Surgical resection is typically performed for the diagnostic purpose or in cases of airway obstruction. Adjuvant radiotherapy following surgery is often recommended to minimize recurrence risk.^[64]^ For tumors >5 cm or those with poor response to radiotherapy, systemic chemotherapy may be necessary.^[65]^ Several factors influence prognosis and recurrence risk, including tumor location, patient’s age, tumor size, histological grade, and bone involvement. Studies have demonstrated that plasmacytomas located outside the paranasal sinuses have a better prognosis. Patients >65 years old, with tumors >5 cm or bone destruction, tend to have poorer outcomes. The involvement of thyroid or cricoid cartilage adversely influences the outcome of solitary extramedullary plasmacytoma of the larynx.^[66]^ In our case, the patient had a small, localized laryngeal lesion without bone involvement, suggesting a favorable prognosis. During 1 year of follow-up, no signs of recurrence or progression were observed. According to recent literature, 5-year survival rate of patients with extramedullary plasmacytoma ranges from 33% to 75%, depending on factors such as age, tumor site, size, and histologic grade. However, local recurrence occurs in approximately 25% of patients, and 15% progress to plasma cell myeloma. By contrast, the 5-year survival rate for plasma cell myeloma is only 18%.^[66]^ Since the increase in Ki-67 expression is associated with the improvement of tumor grade and progression of histological stage, performing Ki-67 immunohistochemical staining may help detect multiple myeloma with potentially aggressive behavior.^[67,68]^ For patients with Ki-67 levels exceeding 50%, it is recommended to undergo adjuvant radiotherapy after surgical resection.^[69]^ Therefore, close long-term follow-up is essential for patients with primary laryngeal plasmacytoma, including clinical assessment, imaging, and laboratory monitoring to detect early signs of systemic involvement.

## Author contributions

**Conceptualization:** Zhong-Shan Luo.

**Formal analysis:** Ming-Hui Wang.

**Resources:** Endi Wang.

**Supervision:** Lian-He Yang.

**Writing – original draft:** Yu-Xin Xia.

**Writing – review & editing:** Lian-He Yang.

## References

[1] Arber DA Orazi A Hasserjian R. The 2016 revision to the World Health Organization classification of myeloid neoplasms and acute leukemia. Blood. 2016;127:2391–405. 27069254 10.1182/blood-2016-03-643544

[2] Dores GM Landgren O McGlynn KA Curtis RE Linet MS Devesa SS. Plasmacytoma of bone, extramedullary plasmacytoma, and multiple myeloma: incidence and survival in the United States, 1992-2004. Br J Haematol. 2009;144:86–94. 19016727 10.1111/j.1365-2141.2008.07421.x PMC2610331

[3] Barbu RR Khan A Port JL Abramson A Gartenhaus WS. Case report: extramedullary plasmacytoma of the larynx. Comput Med Imaging Graph. 1992;16:359–61. 1394084 10.1016/0895-6111(92)90150-8

[4] Mochimatsu I Tsukuda M Sawaki S Nakatani Y. Extramedullary plasmacytoma of the larynx. J Laryngol Otol. 1993;107:1049–51. 8288980 10.1017/s0022215100125241

[5] Weissman JL Myers JN Kapadia SB. Extramedullary plasmacytoma of the larynx. Am J Otolaryngol. 1993;14:128–31. 8484478 10.1016/0196-0709(93)90052-9

[6] Zbären P Zimmermann A. Solitary plasmocytoma of the larynx. ORL J Otorhinolaryngol Relat Spec. 1995;57:50–3. 7700613 10.1159/000276708

[7] Nowak-Sadzikowska J Weiss M. Extramedullary plasmacytoma of the larynx. Analysis of 5 cases. Eur J Cancer. 1998;34:1468. 9849435 10.1016/s0959-8049(98)00063-x

[8] Welsh J Westra WH Eisele D Hogan R Lee DJ. Solitary plasmacytoma of the epiglottis: a case report and review of the literature. J Laryngol Otol. 1998;112:174–6. 9578880 10.1017/s002221510014023x

[9] Rakover Y Bennett M David R Rosen G. Isolated extramedullary plasmacytoma of the true vocal fold. J Laryngol Otol. 2000;114:540–2. 10992939 10.1258/0022215001906093

[10] Nagasaka T Lai R Kuno K Nakashima T Nakashima N. Localized amyloidosis and extramedullary plasmacytoma involving the larynx of a child. Hum Pathol. 2001;32:132–4. 11172308 10.1053/hupa.2001.20896

[11] Kamijo T Inagi K Nakajima M Motoori T Tadokoro K Nishiyama S. A case of extramedullary plasmacytoma of the larynx. Acta Otolaryngol Suppl. 2002;85:104–6. 10.1080/000164802760057707 12212582

[12] Soni NK Trivedi KA Kumar A. Solitary extramedullary plasmacytoma – larynx. Indian J Otolaryngol Head Neck Surg. 2002;54:309–10. 23119921 10.1007/BF02993753 PMC3450465

[13] Nakashima T Matsuda K Haruta A. Extramedullary plasmacytoma of the larynx. Auris Nasus Larynx. 2006;33:219–22. 16406428 10.1016/j.anl.2005.11.019

[14] Velez D Hinojar-Gutierrez A Nam-Cha S Acevedo-Barbera A. Laryngeal plasmacytoma presenting as amyloid tumour: a case report. Eur Arch Otorhinolaryngol. 2007;264:959–61. 17431662 10.1007/s00405-007-0289-x

[15] Kusunoki T Ikeda K Murata K Nishida S Tsubaki M. Extramedullary plasmacytoma of the larynx: a case report from Japan. Ear Nose Throat J. 2007;86:763–4. 18217384

[16] Ozbilen Acar G Yilmaz S Güven Güvenc M Yilmaz M Ozek H Tüziner N. Isolated extramedullary plasmacytoma of the true vocal cord. J Otolaryngol Head Neck Surg. 2008;37:E129–132. 19128655

[17] Grabowska B Grabowski L. [A rare case of plasmocytoma of uvulae and carcinoma of larynx]. Otolaryngol Pol. 2008;62:188–90. 18637444 10.1016/S0030-6657(08)70238-5

[18] Partiba C Sreenivas V Babu M. Plasmacytoma of larynx—a case report. J Voice. 2009;23: 735–8. 18619786 10.1016/j.jvoice.2008.03.009

[19] Vanan I Redner A Atlas M. Solitary extramedullary plasmacytoma of the vocal cord in an adolescent. J Clin Oncol. 2009;27:e244–7. 19858382 10.1200/JCO.2009.23.7461 PMC6340145

[20] Rutherford K Parsons S Cordes S. Extramedullary plasmacytoma of the larynx in an adolescent: a case report and review of the literature. Ear Nose Throat J. 2009;88:E1–7. 19224468

[21] Zhang XL Li DQ Li JJ Li S-S Yang X-M. Synchronous occurrence of extramedullary plasmacytoma and squamous cell carcinoma in situ in the larynx: a case report. Chin J Cancer. 2010;29:1029–34. 21114925 10.5732/cjc.010.10238

[22] Pichi B Terenzi V Covello R Spriano G. Cricoid-based extramedullary plasmocytoma. J Craniofac Surg. 2011;22:2361–3. 22134279 10.1097/SCS.0b013e318231e56d

[23] Kim KS Yang HS Park ES Bae TH. Solitary extramedullary plasmacytoma of the apex of arytenoid: endoscopic, CT, and pathologic findings. Clin Exp Otorhinolaryngol. 2012;5:107–11. 22737292 10.3342/ceo.2012.5.2.107 PMC3380110

[24] González Guijarro I Díez González L Rodriguez Acevedo N Pallas Pallas E. [Extramedullary plasmacytoma of the larynx. A case report]. Acta Otorrinolaringol Esp. 2011;62:320–2. 20511118 10.1016/j.otorri.2010.04.001

[25] Pinto JA Sônego TB Artico MS de Farias Aires Leal C Bellotto S. Extramedullary plasmacytoma of the larynx. Int Arch Otorhinolaryngol. 2012;16:410–3. 25991967 10.7162/S1809-97772012000300019 PMC4432537

[26] Ramírez-Anguiano J Lara-Sánchez H Martínez-Baños D Martínez-Benítez B. Extramedullary plasmacytoma of the larynx: a case report of subglottic localization. Case Rep Otolaryngol. 2012;2012:437264. 23082263 10.1155/2012/437264 PMC3469077

[27] Ravo V Calvanese MG Manzo R. Solitary plasmacytoma of the larynx treated with radiotherapy: a case report. Tumori. 2012;98:35e–8e. 10.1700/1088.11945 22678000

[28] De Zoysa N Sandler B Amonoo-Kuofi K Swamy R Kothari P Mochloulis G. Extramedullary plasmacytoma of the true vocal fold. Ear Nose Throat J. 2012;91:E23–25. 22930090

[29] Ghatak S Dutta M Kundu I Ganguly RP. Primary solitary extramedullary plasmacytoma involving the true vocal cords in a pregnant woman. Tumori. 2013;99:e14–18. 23549014 10.1177/030089161309900126

[30] Loyo M Baras A Akst LM. Plasmacytoma of the larynx. Am J Otolaryngol. 2013;34:172–5. 23312735 10.1016/j.amjoto.2012.11.003

[31] Wang M Du J Zou J Liu S. Extramedullary plasmacytoma of the cricoid cartilage progressing to multiple myeloma: a case report. Oncol Lett. 2015;9:1764–6. 25789038 10.3892/ol.2015.2936 PMC4356401

[32] Xing Y Qiu J Zhou ML. Prognostic factors of laryngeal solitary extramedullary plasmacytoma: a case report and review of literature. Int J Clin Exp Path. 2015;8:2415–35. 26045749 PMC4440058

[33] Pino M Farri F Garofalo P Taranto F Toso A Aluffi P. Extramedullary plasmacytoma of the larynx treated by a surgical endoscopic approach and radiotherapy. Case Rep Otolaryngol. 2015;2015:951583. 26137339 10.1155/2015/951583 PMC4475522

[34] Krebs S Ganly I Ghossein R Yang J Yahalom J Schöder H. Solitary extramedullary plasmacytoma of the cricoid cartilage-case report. Front Oncol. 2017;7:284. 29230383 10.3389/fonc.2017.00284 PMC5711767

[35] Ge S Zhu G Yi Y. Extramedullary plasmacytoma of the larynx: literature review and report of a case who subsequently developed acute myeloid leukemia. Oncol Lett. 2018;16:2995–3004. 30127889 10.3892/ol.2018.8992 PMC6096153

[36] Du YL Yan Y. Solitary extra-medullary plasmacytoma of the true vocal cord. Chin Med J (Engl). 2019;132:1885–6. 31261201 10.1097/CM9.0000000000000303 PMC6759136

[37] Brandt HH Brockmeier SJ Tetter N. Solitary extramedullary plasmacytoma of the larynx: a rare cause of dysphonia. BMJ Case Rep. 2020;13:e234478. 10.1136/bcr-2020-234478 PMC7509959 32963040

[38] Čunović N Košec A Stevanović S Bedeković V. A case report of solitary extramedullary plasmacytoma of the cricoid cartilage diagnosed after total thyroidectomy. Ear Nose Throat J. 2020;99:130–1. 30966804 10.1177/0145561319839634

[39] Ergun U Uysal A Yazici H. Solitary plasmacytoma in a patient presenting with taste disturbance. J Coll Physicians Surg Pak. 2022;32:S165–7. 36210683 10.29271/jcpsp.2022.Supp2.S165

[40] Lu G Zhang Q. Extramedullary plasmacytoma of false vocal cord: case report. Ear Nose Throat J. 2022;101:NP348–50. 33155846 10.1177/0145561320971929

[41] Shankar R Singh R Nanda SS Tripathi P Mishra A. Extramedullary solitary plasmacytoma of larynx: a rare entity and therapeutic challenge. Indian J Otolaryngol Head Neck Surg. 2022;74(Suppl 3):5122–6. 36742488 10.1007/s12070-021-02910-4 PMC9895687

[42] Tanrivermis Sayit A Elmali M Gün S. Evaluation of extramedullary plasmacytoma of the larynx with radiologic and histopathological findings. Radiologia (Engl Ed). 2022;64:69–73. 35180989 10.1016/j.rxeng.2020.07.007

[43] Miyamori M Ichikawa T Inamura N. Primary lymphoplasmacytic lymphoma of the larynx mimicking extramedullary plasmacytoma: a case report. Oncol Lett. 2024;27:132. 38362232 10.3892/ol.2024.14265 PMC10867733

[44] Hodroj H Saker Z Al Najjar A Choukr H El Moussaoui MRN. Laryngeal plasmacytoma in a patient with Down’s syndrome. Autops Case Rep. 2024;14:e2024508. 39176104 10.4322/acr.2024.508 PMC11340819

[45] Gupte A Sasidharan A Dutta D Anoop R. Extramedullary plasmacytoma of the larynx - case report. J Cancer Res Ther. 2024;20:493–5. 38554374 10.4103/jcrt.JCRT_1640_20

[46] Fend F Dogan A Cook JR. Plasma cell neoplasms and related entities-evolution in diagnosis and classification. Virchows Arch. 2023;482:163–77. 36414803 10.1007/s00428-022-03431-3 PMC9852202

[47] Brigle K Rogers B. Pathobiology and diagnosis of multiple myeloma. Semin Oncol Nurs. 2017;33:225–36. 28688533 10.1016/j.soncn.2017.05.012

[48] Bailly J Jenkins N Chetty D Mohamed Z Verburgh ER Opie JJ. Plasmablastic lymphoma: an update. Int J Lab Hematol. 2022;44:54–63. 36074710 10.1111/ijlh.13863 PMC9545967

[49] Li JW Peng HL Zhou XY Wang J-J. Plasmablastic lymphoma: current knowledge and future directions. Front Immunol. 2024;15:1354604. 38415257 10.3389/fimmu.2024.1354604 PMC10896986

[50] Patil PA DeLellis RA. Plasma cell granuloma of the thyroid: review of an uncommon entity. Arch Pathol Lab Med. 2018;142:998–1005. 30040458 10.5858/arpa.2017-0068-RS

[51] Xiang H Wu Z Wang Z Yao H. Nodular pulmonary amyloidosis and obvious ossification due to primary pulmonary MALT lymphoma with extensive plasmacytic differentiation: report of a rare case and review of the literature. Int J Clin Exp Pathol. 2015;8:7482–7. 26261657 PMC4525991

[52] Aggarwal S Kaushal V Singla S Sen R. Primary glottic malignant melanoma of the larynx (PGMML): a very rare entity. BMJ Case Rep. 2015;2015:bcr2015211317. 10.1136/bcr-2015-211317 PMC4680593 26590185

[53] Zhou L Zhou EH. Laryngeal primary myoepithelial carcinoma: case report and a systematic review of literature. Ear Nose Throat J. 2023;17:1455613231165156. 10.1177/01455613231165156 36931828

[54] Yu G Qu G Kong L Pan X Wang W Lv J. Primary myoepithelial carcinoma of the larynx: case report and review of the literature. Pathol Res Pract. 2011;207:127–30. 21109359 10.1016/j.prp.2010.10.006

[55] Rajkumar SV Dimopoulos MA Palumbo A. International Myeloma Working Group updated criteria for the diagnosis of multiple myeloma. Lancet Oncol. 2014;15:e538–48. 25439696 10.1016/S1470-2045(14)70442-5

[56] Plasma Cell Disease Group, Chinese Society of Hematology, Chinese Medical Association; Chinese Myeloma Committee-Chinese Hematology Association. [Consensus for the diagnosis and management of extramedullary plasmacytoma in China (2024)]. Zhonghua Xue Ye Xue Za Zhi. 2024;45:8–17. 38527832 10.3760/cma.j.cn121090-20231107-00253 PMC10951115

[57] Zhang J Ding D Sun J. A case report of multiple extramedullary plasmacytoma of the head and neck. Medicine (Baltimore). 2022;101:e32203. 36482521 10.1097/MD.0000000000032203 PMC9726292

[58] Huang L Wei J Wang F. Epidemiology and survival of primary extraosseous plasmacytoma: insights from a population-based study with a 20-year follow-up. Leuk Lymphoma. 2023;64:2026–36. 37584346 10.1080/10428194.2023.2245512

[59] Finsinger P Grammatico S Chisini M Piciocchi A Foà R Petrucci MT. Clinical features and prognostic factors in solitary plasmacytoma. Br J Haematol. 2016;172:554–60. 26684545 10.1111/bjh.13870

[60] Susnerwala SS Shanks JH Banerjee SS Scarffe JH Farrington WT Slevin NJ. Extramedullary plasmacytoma of the head and neck region: clinicopathological correlation in 25 cases. Br J Cancer. 1997;75:921–7. 9062417 10.1038/bjc.1997.162 PMC2063399

[61] Yan B Tan SY Yau EX Ng SB Petersson F. EBV-positive plasmacytoma of the submandibular gland—report of a rare case with molecular genetic characterization. Head Neck Pathol. 2011;5:389–94. 21442194 10.1007/s12105-011-0257-z PMC3210227

[62] Mock PM Neal GD Aufdemorte TB. Immunoperoxidase characterization of extramedullary plasmacytoma of the head and neck. Head Neck Surg. 1987;9:356–61. 3305425 10.1002/hed.2890090610

[63] Goyal G Bartley AC Funni S. Treatment approaches and outcomes in plasmacytomas: analysis using a national dataset. Leukemia. 2018;32:1414–20. 29654264 10.1038/s41375-018-0099-8 PMC6522261

[64] Ghiassi-Nejad Z Ru M Moshier E Chang S Jagannath S Dharmarajan K. Overall survival trends and clinical characteristics of plasmacytoma in the United States: a national cancer database analysis. Clin Lymphoma Myeloma Leuk. 2019;19:310–9. 30878315 10.1016/j.clml.2019.01.004 PMC6555681

[65] Caers J Paiva B Zamagni E. Diagnosis, treatment, and response assessment in solitary plasmacytoma: updated recommendations from a European Expert Panel. J Hematol Oncol. 2018;11:10. 29338789 10.1186/s13045-017-0549-1 PMC5771205

[66] Szczepanek E Drozd-Sokołowska J Sokołowski J. Solitary extramedullary plasmacytoma of the larynx and secondary laryngeal involvement in plasma cell myeloma: single-centre retrospective analysis and systematic literature review. J Clin Med. 2022;11:4390. 35956004 10.3390/jcm11154390 PMC9369432

[67] Kanavaros P Stefanaki K Vlachonikolis J. Immunohistochemical expression of the p53, p21/Waf-1, Rb, p16 and Ki67 proteins in multiple myeloma. Anticancer Res. 2000;20:4619–25. 11205312

[68] Steensma DP Gertz MA Greipp PR. A high bone marrow plasma cell labeling index in stable plateau-phase multiple myeloma is a marker for early disease progression and death. Blood. 2001;97:2522–3. 11290618 10.1182/blood.v97.8.2522

[69] Wei X Shen J Zheng S. Extramedullary plasmacytoma of the Gingiva: a rare case report and clinical management. Oral Oncol. 2025;165:107315. 40306237 10.1016/j.oraloncology.2025.107315

